# Impact of bistrand abasic sites and proximate orientation on DNA global structure and duplex energetics

**DOI:** 10.1002/bip.23098

**Published:** 2018-01-11

**Authors:** Conceição A. Minetti, Jeffrey Y. Sun, Daniel P. Jacobs, Inkoo Kang, David P. Remeta, Kenneth J. Breslauer

**Affiliations:** ^1^ Department of Chemistry and Chemical Biology Rutgers ‐ The State University of New Jersey Piscataway New Jersey

**Keywords:** bistrand abasic sites, clustered lesions, DNA damage, thermodynamic stability

## Abstract

Bistrand lesions embedded within a single helical turn of tridecameric deoxyoligonucleotide duplexes represent a model system for exploring the impact of clustered lesions that occur in vivo and pose a significant challenge to cellular repair machineries. Such investigations are essential for understanding the forces that dictate lesion‐induced mutagenesis, carcinogenesis, and cytotoxicity within a context that mimics local helical perturbations caused by an ionizing radiation event. This study characterizes the structural and energy profiles of DNA duplexes harboring synthetic abasic sites (tetrahydrofuran, F) as models of clustered bistrand abasic (AP) lesions. The standard tridecameric dGCGTACCCATGCG·dCGCATGGGTACGC duplex is employed to investigate the energetic impact of single and bistrand AP sites by strategically replacing one or two bases within the central CCC/GGG triplet. Our combined analysis of temperature‐dependent UV and circular dichroism (CD) profiles reveals that the proximity and relative orientation of AP sites within bistrand‐damaged duplexes imparts a significant thermodynamic impact. Specifically, 3′‐staggered lesions (CCF/GFG) exert a greater destabilizing effect when compared with their 5′‐counterpart (FCC/GFG). Moreover, a duplex harboring the central bistrand AP lesion (CFC/GFG) is moderately destabilized yet exhibits distinct properties relative to both the 3′ and 5′‐orientations. Collectively, our energetic data are consistent with structural studies on bistrand AP‐duplexes of similar sequence in which a 3′‐staggered lesion exerts the greatest perturbation, a finding that provides significant insight regarding the impact of orientation on lesion repair processing efficiency.

## 1 INTRODUCTION

Oxidative damage in DNA has been associated with aging and a host of ailments including chronic inflammation, degenerative autoimmune diseases, and cancer.^1^ The presence of damaged sites in DNA is an important determinant in the overall health of a cell. Despite the fact that one mammalian cell may accumulate thousands of lesions (e.g., nearly 20,000 8‐hydroxyguanine sites) per day,^2^ the vigilance of highly efficient DNA damage repair systems processes these lesions effectively. As the cellular repair efficiency declines with aging, the levels of damaged sites progressively increases and there is a greater risk for age‐related diseases including cancer.^3^ One particular damage of considerable interest is the abasic (apurinic/apyrimidinic) AP site, which constitutes an intermediate/product of most DNA damage processing pathways and occurs with high frequencies in malignant tumors and/or following ionizing irradiation.^4^

Studies on AP‐site containing duplexes employing biophysical and biochemical approaches have provided specific details on the fate of a lesion within the cell.^5^, ^6^ Moreover, these investigations have revealed the impact of base sequence, conformation, and a number of other properties that play a critical role on the resultant outcomes.^7^, ^8^, ^9^, ^10^ While insightful, the majority of these studies have used site‐specific AP‐damaged duplexes [as reviewed in Ref. 11] and relatively few explore the impact of clustered AP‐lesions that represents a more common scenario in vivo. The latter are a direct consequence of internal and external chemical assaults arising from metabolic sources, UV radiation, and carcinogens that create reactive oxygen species. The locally multiply damaged sites (LMDS) or clustered lesions are defined as two or more lesions occurring within a short oligonucleotide stretch.^12^ During ionizing radiation or radiomimetic anticancer treatments, two or more lesions appear frequently as clusters, either in the same or opposing strands within a single turn of the DNA helix.^13^, ^14^, ^15^, ^16^, ^17^, ^18^ It is therefore relevant to investigate the impact of LMDS, their relative distance and orientation on DNA duplex conformation and stability, and the consequences regarding recognition and repair.

In general, LMDS are considered to be more genotoxic or mutagenic than a single isolated lesion. Biochemical and biological studies have demonstrated that clustered lesions are less repairable than their individual counterparts, and the mutation of one lesion is synergized by the presence of another within its immediate vicinity.^19^ Conversely, the efficiency of clustered lesion excisions may also result in challenging scenarios to the cell as these often generate double strand breaks (as reviewed in Ref. 19). Incompletely or incorrectly repaired lesions are generally presumed responsible for the cytotoxic effects of ionizing radiation. Due to the proximity of damage formation, LMDS are repair‐resistant with high mutagenic potential.^19^ Studies on synthetic deoxyoligonucleotides have suggested that mutagenic potential is inversely proportional to the spacing between lesions. Repair resistant clustered lesions are persistent and may therefore remain for significant periods of time following irradiation. The repair efficiency of clustered oxidative damage has been reported to depend on its nature, sequence context, and relative orientation.^12^, ^18^, ^20^, ^21^

This study is part of a systematic investigation to evaluate lesion impacts on DNA duplex energetics. Such studies contribute to our expanding thermodynamic database of nucleic acid modifications, which are essential for understanding the forces that dictate lesion‐induced mutagenesis, carcinogenesis, and cytotoxicity. Our immediate aim is to characterize the energy profiles of DNA duplexes harboring one or two proximately spaced bistrand AP‐sites employing the synthetic analog tetrahydrofuran (F). The latter has been widely employed as a suitable site‐specific abasic analog in biophysical studies.^7^, ^8^, ^9^, ^10^, ^11^ Moreover, duplexes harboring this synthetic abasic nucleoside bind repair enzymes (e.g., Fpg) with high affinity,^22^ are substrates for APE endonucleases,^23^ and inhibit glycosylase‐mediated 8‐oxodeoxyguanosine base excision.^24^ A broader goal is to correlate the energetic data with biochemical and structural studies. These considerations necessitate the acquisition of measurements under solution conditions that approximate the physiological cellular environment. The combined analysis of UV and circular dichroism (CD) profiles reveals that the relative orientation of AP sites in bistrand‐damaged duplexes imparts a significant energetic impact. Specifically, 3′‐staggered lesions exert a greater destabilizing effect when compared to their 5′‐counterparts while a central bistrand lesion exhibits a moderately destabilizing effect that is distinguishable from both the 3′ and 5′‐orientations. Such studies contribute to an expanding thermodynamic database of nucleic acid modifications and are a critical component of our overall objective to characterize DNA duplexes harboring lesions of biological and toxicological relevance.

## 2 MATERIALS AND METHODS

### 2.1 Deoxyoligonucleotide synthesis, purification, and hybridization

**Scheme 1:**
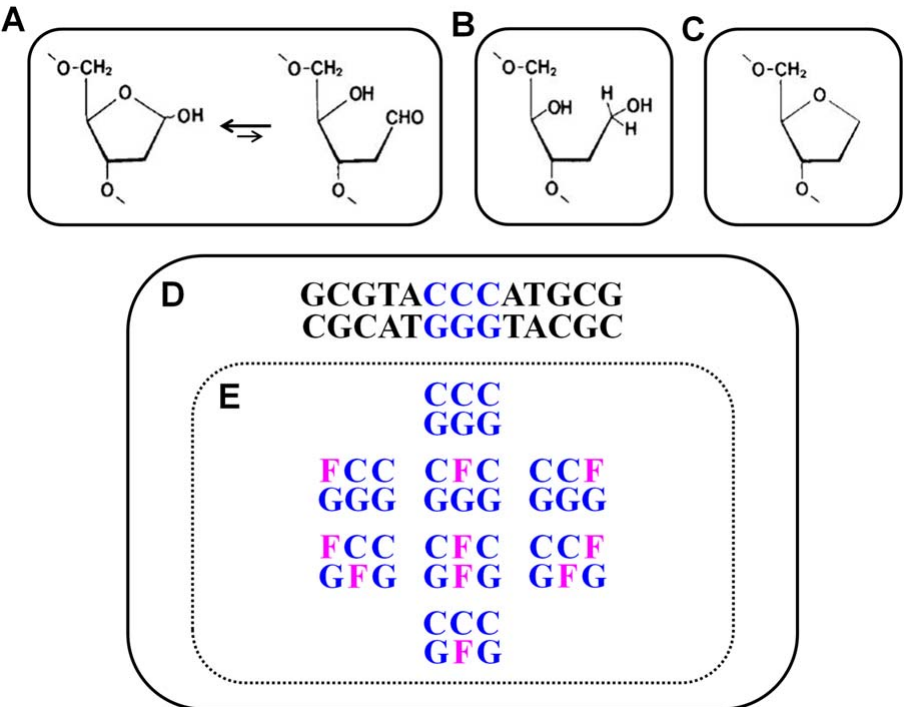
Representation of the Eight Tridecameric Duplexes Studied Herein. Chemical structures of AP‐sites: (A) Natural; (B) Reduced; and, (C) Synthetic, F (tetrahydrofuran) [based on Ref. 64]. D, Parent tridecamer duplex comprised of a central CCC/GGG triplet. E, Central triplet designating the series of tridecameric duplexes characterized in this study including Watson‐Crick parent (CCC/GGG); single strand abasic sites (**F**CC/GGG, C**F**C/GGG, and CC**F**/GGG); bistrand abasic sites (5′‐staggered **F**CC/G**F**G, central C**F**C/G**F**G, and 3′‐staggered CC**F**/G**F**G); and, single abasic site (CCC/G**F**G) employed as a reference for bistrand‐AP duplexes

Synthesis of the undamaged tridecanucleotide sequences d(GCGTACCCATGCG) and d(CGCATGGGTACGC) were performed using standard phosphoramidite chemistry. AP‐lesions were embedded as a single residue at various positions within the central CCC/GGG triplet employing specific THF phosphoramidites. The tridecameric deoxyoligonucleotides were purified via a combination of reverse phase and anion exchange chromatography as described previously.^25^, ^26^ The identity and integrity of the single and bistrand AP‐site oligonucleotides were assessed by mass spectrometry. A total of six deoxyoligonucleotides were synthesized and eight duplexes hybridized to yield five single AP and three bistrand AP‐duplexes as illustrated in Scheme 1. Molar extinction coefficients were determined as described elsewhere^27^ and confirmed via the method of continuous fractions^28^, ^29^ in which the AP‐containing strands are annealed with complementary lesion‐free strands to form the single lesion‐duplexes. All of the experiments employed a buffer system comprised of 10 mM sodium phosphate and 150 mM NaCl adjusted to pH 7.0.

### 2.2 CD spectroscopy

CD spectropolarimetry was employed to evaluate the global structure of each duplex. Spectra were acquired between 200 and 350 nm at 1.0 nm resolution and 15 s averaging on an Aviv Model 420 CD spectropolarimeter (Aviv Biomedical, Lakewood, NJ) using a 1 mm quartz cuvette and total DNA strand concentration of 45 μM. The CD spectra were buffer subtracted, smoothed using the Savitzky‐Golay algorithm,^30^ and concentration normalized to yield molar ellipticity defined as 100 θ/*cl*,^31^ where θ is ellipticity (degrees), *c* is duplex concentration (M), and *l* is pathlength (cm). Temperature‐dependent CD profiles were recorded at 5.0°C increments over the range of 0 to 95.0°C to monitor the thermal induced disruption of duplex global structure. The molar ellipticity at 250 nm is plotted as a function of temperature to yield thermal melting profiles of the respective duplexes for comparison with temperature‐dependent UV data acquired at 260 nm.

### 2.3 Temperature‐dependent optical methods

Temperature‐dependent UV absorbance profiles were acquired for each duplex at a minimum of five concentrations spanning the range 0.5–50 μM total strand concentration (*C*_T_). Samples in quartz cuvettes (0.1–1.0 cm pathlength) were heated in the thermostatted sample compartment of an Aviv Model 14 UV/Vis spectrophotometer (Aviv Biomedical, Lakewood, NJ) over the temperature range of 0–95°C. The absorbance at 260 nm was recorded at 0.5°C increments following integration for 15 s to monitor the hyperchromicity change as a function of temperature. The duplex melting temperatures (*T*_m_) and van't Hoff dissociation thermodynamic parameters have been determined via shape analysis of the melting profiles assuming a zero net change in heat capacity as described previously.^32^ The van't Hoff enthalpies and entropies are also determined from a series of concentration‐dependent UV melting profiles in which the reciprocal of *T*_m_ is plotted versus the log of DNA concentration as described previously.^32^ Analysis of the resultant slopes and intercepts yields the dissociation enthalpy and entropy for each duplex via application of the following relation: *1/T*_m_ *= (R/*Δ*H°)* ln *C_T_* + (Δ*S° – R* ln 4)/Δ*H°*. The free energy change (Δ*G°*) characterizing duplex dissociation at a desired temperature (*T*) is determined from the relation: Δ*G°* = Δ*H*° (1 – *T*/*T*_m_) – *RT* ln (*C*_T_/4). The derivation of van't Hoff dissociation enthalpies (Δ*H*°_vH_) via multiple approaches affords evaluation of the two‐state unfolding behavior.^33^ A comparative analysis of the ratio between Δ $H_{\text{vH}}^{\text{conc}}$ and Δ $H_{\text{vH}}^{\text{shape}}$ yields an estimate for the melting cooperativity thereby assessing the validity of applying a two‐state approach.

## 3 RESULTS

### 3.1 Experimental strategy

Studies focused on the impact of LMDS are relatively scarce when compared with those that explore the thermodynamic consequences of single base damages in DNA. This project aims at furthering investigations on the energetics of duplexes harboring two proximate lesions and the impact of their relative orientation within a cluster. As model systems for the highly reactive and unstable naturally occurring AP‐site (Scheme 1A), in vitro studies employ synthetic variants such as a reduced abasic site (Scheme 1B) and tetrahydrofuran, F (Scheme 1C). In our study, the synthetic AP‐analog (F) is incorporated at defined positions within a tridecameric deoxyoligonucleotide duplex (Scheme 1D) to mimic the naturally occurring bistrand AP sites. Specifically, we evaluate the impact of F‐sites positioned within the central triplet (CCC/GGG) as a single or bistrand lesion at various orientations as illustrated in Scheme 1E. While the nomenclature varies somewhat amongst different laboratories,^14^, ^21^, ^34^ we adopt a consensus in which the 5′‐staggered or 5′‐orientation = + *n*, and the 3′‐staggered or 3′‐orientation = – *n*, where *n* represents the number of base pairs separating both lesions in opposite strands.^21^ This decision is based solely on the need to facilitate structural‐energetics correlations given the availability of published NMR studies on identical sequences.^21^ The designation of *n*+/–1 base‐pairs apart applies to **F**CC/G**F**G or CC**F**/G**F**G, whereas *n* = 0 refers to the C**F**C/G**F**G bistrand duplex. Our systematic investigation of AP‐bistrand impacts on DNA duplex energetics employs temperature‐dependent UV and CD spectroscopy.

### 3.2 Global structural analysis via CD spectroscopy

**Figure 1:**
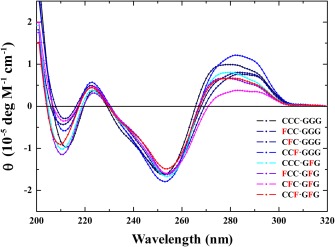
CD Profiles for the Eight Tridecameric Duplexes Characterized Herein. Spectra for the family of eight DNA duplexes acquired at 0°C are designated by their respective acronyms and color‐coded as indicated. Damaged duplexes harboring a single AP‐site in the top strand (**F**CC/GGG, C**F**C/GGG, and CC**F**/GGG) are characterized by ellipticity maxima at ∼280 nm, while a modest blue shift is observed for duplexes containing an abasic site in the bottom strand (CCC/G**F**G, **F**CC/G**F**G, C**F**C/G**F**G, and CC**F**/G**F**G)

We employed CD spectroscopy to evaluate changes in duplex global structure and conformation. The family of CD spectra presented in Figure 1 illustrates the impact of single and bistrand abasic site lesions on DNA duplex structure. These deoxyoligonucleotides exhibit specific characteristics that are typical of B‐DNA including a positive ellipticity in the wavelength range of 270–290 nm and a negative band at 250 nm with an amplitude that is sequence‐dependent.^35^ While the CD spectra reveal significant differences due to the presence of AP sites, the expected right handed conformation is retained in all duplexes. Specifically, inspection of the CD spectra acquired at 0°C for these duplexes reveals that introduction of a single or bistrand lesion does not significantly disrupt the typical B‐DNA conformation.

Contributions arising from the spatial orientation of each base chromophore per se affects the magnitude of ellipticity, as the latter also depends on base composition and sequence.^8^ These impacts are normalized at least in part when comparing subgroups of single and bistrand AP‐site duplexes. Despite measureable changes in ellipticity magnitude and/or wavelength minima/maxima, the family of CD spectra presented in Figure 1 reveals that these eight duplexes adopt an overall B‐DNA conformation. As a reference for the C**F**C/G**F**G duplex, we recorded the CD spectrum of a dodecamer containing an identical sequence as documented in previous studies.^7^ The two duplexes exhibit comparable profiles with a slight reduction of ellipticity observed for the bistrand relative to undamaged dodecamer in the 270–280 nm wavelength region.

### 3.3 Temperature‐dependent CD studies

**Figure 2:**
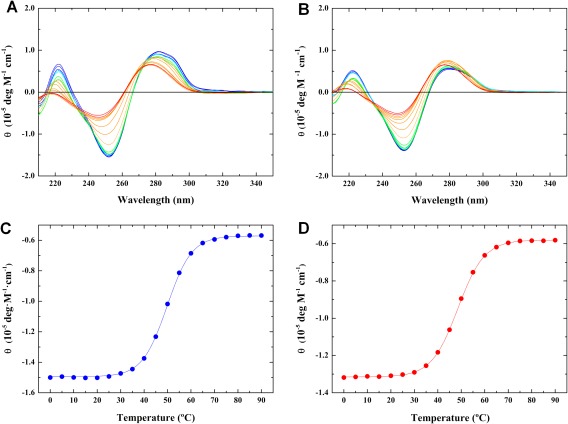
Representative Temperature‐Dependent CD Profiles for the Single AP‐Site C**F**C/GGG (Panels A, C) and Bistrand AP‐Site C**F**C/G**F**G (Panels B, D) Duplexes. The CD spectra (Panels A and B) are acquired at 5.0°C intervals over the temperature range of 0–90°C. Molar ellipticities at 250 nm are plotted as a function of temperature (Panels C and D) to derive duplex dissociation transition temperatures of 49.0 and 47.1°C for C**F**C/GGG and C**F**C/G**F**G, respectively

We monitored the temperature‐dependent stability of DNA global structure as depicted in representative CD profiles of duplexes harboring single (C**F**C/GGG) and bistrand (C**F**C/G**F**G) abasic sites. Panels A and B in Figure 2 present profiles recorded at 5°C increments, while panels C and D are plots of the molar ellipticities monitored at 250 nm as a function of temperature. The temperature‐dependent decrease in molar ellipticity at 250 nm parallels changes in the hyperchromicity derived from temperature‐dependent UV measurements, yielding transition temperatures (*T*_m_) that are virtually indistinguishable for the respective duplexes studied at identical concentrations. A shape analysis of the CD profiles reveal comparable model‐dependent thermodynamic parameters.

### 3.4 van't Hoff analysis of optical melting profiles

As part of our systematic effort to evaluate the impact of abasic lesions on the thermal and thermodynamic properties of duplex DNA, we performed a shape analysis of the temperature‐induced dissociation profiles for direct comparison with the concentration dependence of transition temperatures (Refer to Materials and Methods). Although both methods provide useful information regarding model‐dependent thermodynamic properties, we specifically selected the latter to characterize AP‐lesion impacts since it provides a more robust evaluation of the energetic parameters by enveloping a wide range (i.e., 0.8–45 μM) of total strand concentrations^25^, ^26^, ^36^, ^37^, ^38^ and avoids potential uncertainties associated with baseline assignments/extrapolations that might compromise the shape analysis method.^37^ Despite our reliance on the concentration‐dependent approach, we employed both methods to assess the overall cooperativity of duplex dissociation.^33^ While van't Hoff analysis is widely used to characterize macromolecular processes, it is important to recognize that this method is only valid for two‐state processes. Model‐independent calorimetric approaches (i.e., DSC and ITC) are required for a complete thermodynamic characterization of the duplex to single strand association/dissociation process and verification of its two‐state nature. In the absence of calorimetric data, the combined application of two independent optical approaches provides insight regarding the impact of lesions on thermodynamic parameters as well as the two‐state nature of duplex dissociation.^33^

A family of normalized optical melting profiles monitoring the temperature‐induced dissociation of duplexes in the absence and presence of AP‐sites has been acquired. Eight sets of temperature‐dependent UV profiles representing a minimum of five different duplex concentrations provide a complete characterization of thermodynamic parameters. DNA duplex transition temperatures (*T*_m_) are determined via shape analysis of the absorbance profiles acquired at each concentration. The duplex transition temperatures are cast as a function of concentration, where the reciprocal of *T*_m_ is plotted versus the natural logarithm of total strand concentration. As deduced from these reciprocal plots, the duplex dissociation enthalpy (Δ*H*) and entropy (Δ*S*) are derived from the slopes and *y*‐intercepts, respectively. The van't Hoff thermodynamic parameters determined via analysis of concentration‐dependent duplex dissociation profiles are summarized in Table 1. The resultant data compare favorably with shape analysis of temperature‐induced dissociation profiles presented in Supporting Information Table S1.

**Table 1:** van't Hoff thermodynamic parameters derived via a combination of concentration‐dependent and shape analysis of temperature‐dependent melting profiles acquired for the eight tridecameric duplexes studied herein

Acronym	*T*_m_ (°C)	Δ*H*°_conc_ (kcal mol^−1^)	*T*Δ*S*°_conc_ (kcal mol^−1^)	Δ*G*°_conc_ (kcal mol^−1^)	Δ*H*°_shape_ (kcal mol^−1^)	Δ*H*°_shape_/Δ*H*°_conc_
**CCC/GGG**	65.2 ± 0.1	110.6 ± 0.1	91.6 ± 0.9	19.0 ± 0.3	113.7 ± 0.1	1.0 ± 0.01
**FCC/GGG**	48.7 ± 0.2	99.1 ± 0.2	85.9 ± 0.2	13.2 ± 0.3	69.9 ± 1.7	0.7 ± 0.02
**CFC/GGG**	48.2 ± 0.2	92.3 ± 0.9	79.8 ± 1.0	12.4 ± 0.4	86.7 ± 0.5	0.9 ± 0.01
**CCF/GGG**	48.3 ± 0.2	94.2 ± 1.3	81.4 ± 1.3	12.8 ± 0.3	70.8 ± 0.9	0.8 ± 0.03
**CCC/GFG**	48.3 ± 0.2	90.7 ± 1.3	78.2 ± 1.1	12.5 ± 0.3	79.0 ± 1.5	0.9 ± 0.04
**FCC/GFG**	48.9 ± 0.1	79.3 ± 0.1	67.4 ± 0.9	11.9 ± 0.4	85.8 ± 2.5	1.1 ± 0.05
**CFC/GFG**	46.4 ± 0.1	90.9 ± 0.2	79.0 ± 0.4	11.9 ± 0.3	81.9 ± 2.0	0.9 ± 0.05
**CCF/GFG**	44.9 ± 0.2	74.1 ± 1.5	63.3 ± 1.5	10.7 ± 0.3	77.0 ± 2.0	1.0 ± 0.03

Transition temperatures correspond to a total strand concentration (*C*_T_) of 45.4 μM. Thermodynamic parameters are reported at 25°C assuming Δ*C*_p_ = 0. Data correspond to averages and standard errors for a minimum of three independent experiments. Melting cooperativity deduced from the ratio of van't Hoff dissociation enthalpies calculated via shape analysis and concentration dependence of the thermal dissociation profiles.

### 3.5 AP lesion impacts on DNA duplex energetics

Inspection of the concentration‐dependent data reveals that all of the AP‐duplexes are significantly destabilized relative to the parent tridecamer duplex. As illustrated in Table 1, the introduction of a single AP lesion reduces the enthalpy of duplex dissociation (Δ*H*) by a substantial factor. Specifically, the presence of a single lesion enthalpically destabilizes and entropically stabilizes the duplex by an average of 16.6 and 10.3 kcal mol^−1^, respectively. The concomitant reduction in dissociation free energy averages 6.3 kcal mol^−1^. Duplexes containing two AP lesions exhibit additional enthalpic destabilization with the notable exception of C**F**C/G**F**G in which the presence of a second lesion does not appreciably reduce the measured dissociation enthalpy relative to a single AP‐site. Likewise, all of the AP‐duplexes exhibit lower unfavorable association entropies (*T*Δ*S*) relative to the parent tridecamer. In this particular case, bistrand damaged duplexes exhibit an even lower unfavorable entropic contribution with the exception of a central bistrand duplex (C**F**C/G**F**G). The reduction in ΔG of single site damaged duplexes is magnified further for bistrand lesions, amongst which the CC**F**/G**F**G duplex is particularly destabilized relative to **F**CC/G**F**G and C**F**C/G**F**G.

### 3.6 Differential impact of AP lesions on DNA duplex thermal and thermodynamic stability

An improved visualization of AP lesion impacts may be gleaned from systematic evaluation of the differential thermal (Δ*T*_m_) and thermodynamic destabilization energies (i.e., ΔΔ*H*, Δ*T*Δ*S*, and ΔΔ*G*) relative to the undamaged parent tridecamer duplex as reported in Table 2. The values of Δ*T*_m_, ΔΔ*H*, Δ*T*Δ*S*, and ΔΔ*G* are calculated by subtracting the respective dissociation parameter for each damaged duplex from that of the parent duplex, all of which are derived from concentration‐dependent plots. Inspection of the data in Table 2 reveals that all of the duplexes studied exhibit a significant reduction in transition temperature (Δ*T*_m_ ∼ 16 – 20°C), dissociation enthalpy (ΔΔ*H* = 11.4 – 36.6 kcal mol^−1^), dissociation entropy (Δ*T*Δ*S* = 5.7 – 28.3 kcal mol^−1^), and dissociation free energy (ΔΔ*G* = 5.8 – 8.2 kcal mol^−1^). The thermal and thermodynamic parameters with their corresponding uncertainties and statistical significance are cast in the form of histograms as illustrated in Supporting Information Figures S1–S4.

**Table 2:** Comparison of thermodynamic impacts for: (A) single abasic damaged relative to the Watson‐Crick parent duplex; (B) bistrand abasic damaged relative to the Watson‐Crick parent duplex; (C) bistrand abasic relative to bottom strand‐damaged duplex; (D) bistrand abasic relative to top strand‐damaged duplex

Acronym	Δ*T*_m_ (°C)	ΔΔ*H*° (kcal mol^−1^)	Δ*T*Δ*S*° (kcal mol^−1^)	ΔΔ*G*° (kcal mol^−1^)
Single Abasic Relative to Parent Duplex				
**FCC/GGG**	−16.5	−11.5	−5.7	−5.8
**CFC/GGG**	−17.0	−18.3	−11.8	−6.5
**CCF/GGG**	−16.9	−16.4	−10.2	−6.2
**CCC/GFG**	−16.9	−19.9	−13.4	−6.5
Bistrand Abasic Relative to Parent Duplex				
**FCC/GFG**	−16.3	−31.3	−24.2	−7.1
**CFC/GFG**	−18.8	−19.7	−12.6	−7.1
**CCF/GFG**	−20.3	−36.5	−28.3	−8.2
Bistrand Abasic Relative to Bottom Strand Damaged Abasic Site				
**FCC/GFG**	+0.6	−11.4	−10.8	−0.6
**CFC/GFG**	−1.9	+0.2	+0.8	−0.6
**CCF/GFG**	−3.4	−16.6	−14.9	−1.7
Bistrand Abasic Relative to Top Strand Damaged Abasic Site				
**FCC/GFG**	+0.2	−19.8	−18.5	−1.3
**CFC/GFG**	−1.8	−1.4	−0.8	−0.6
**CCF/GFG**	−3.4	−20.1	−18.1	−2.0

### 3.7 Energetic impact of a single abasic site at various positions within the central triplet

Introduction of a single abasic site within the central triplet thermally destabilizes a parent tridecamer duplex by 16.8 ± 0.2°C irrespective of lesion position. Despite the comparable transition temperatures of these damaged duplexes, we observe a differential energetic impact that is dependent on lesion position. Specifically, a single AP‐site enthalpically destabilizes the duplexes (ΔΔ*H* = 11.5 – 19.9 kcal mol^−1^), which are ranked as follows: C**F**C ∼ G**F**G ≥ CC**F** > **F**CC. In terms of the entropic contribution, a single AP‐site stabilizes the duplexes (Δ*T*Δ*S* = 5.7 – 13.5 kcal mol^−1^), which are ranked as follows: G**F**G ∼ C**F**C ≥ CC**F** > **F**CC. Consequently, incorporation of a single AP‐site thermodynamically destabilizes the parent tridecamer duplex with ΔΔ*G* values spanning the range of 5.8–6.5 kcal mol^−1^.

### 3.8 Energetic impact of bistrand abasic sites at different orientations within the central triplet

Our data reveal that the presence of two abasic sites imparts additional duplex destabilization, which is a function of their relative orientation. The 3′‐staggered lesion has a greater destabilizing effect for both the dissociation enthalpy and free energy when compared to the 5′‐staggered lesion. Consequently, the most destabilized duplex corresponds to the 3′‐staggered bistrand (CC**F**/G**F**G) in which an enhanced reduction of free energy (ΔΔ*G* = 8.2 kcal mol^−1^) may be attributed to a combined enthalpic decrease and entropic increase. Interestingly, despite the lower enthalpic reduction of a central bistrand‐AP (C**F**C/G**F**G), we observe comparable thermodynamic destabilization (ΔΔ*G* = 7.1 kcal mol^−1^) for the 5′‐staggered duplex (**F**CC/G**F**G) due to a less favorable dissociation entropy.

**Figure 3:**
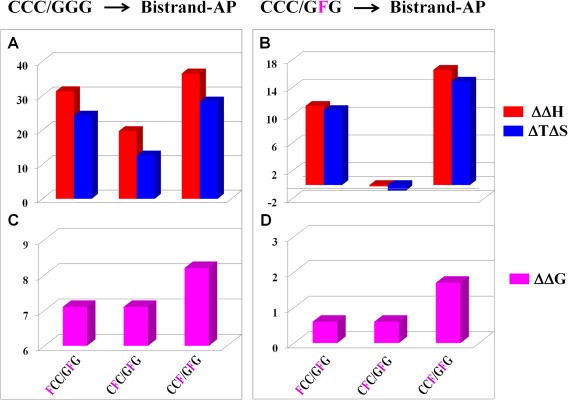
Energetic Impact of Introducing a Second AP‐Site within CCC/G**F**G at the 5′‐staggered (**F**CC/GFG), 3′‐staggered (CC**F**/G**F**G), and Central (C**F**C/G**F**G) Orientations, respectively. Effect of introducing two abasic sites within the canonical undamaged duplex (Left Panel) or a second abasic site within a single AP‐site duplex (Right Panel). Differential data are derived by subtracting dissocition parameters from the corresponding reference (CCC/GGG) and single AP‐site (CCC/G**F**G) duplexes. The bistrand abasic site duplexes are thermodynamically destabilized relative to the undamaged parent tridecamer with CC**F**/G**F**G > **F**CC/G**F**G ∼ C**F**C/G**F**G (Left Panel). Further destabilization is observed for the 3′staggered bistrand duplex relative to CCC/G**F**G with CC**F**/G**F**G > **F**CC/G**F**G ∼ C**F**C/G**F**G (Right Panel)

Relative to the parent tridecamer duplex, introduction of bistrand AP‐sites at different orientations within the central triplet are enthalpically destabilizing (ΔΔ*H* = 19.7 – 36.5 kcal mol^−1^) and entropically stabilizing (Δ*T*Δ*S* = 12.7 – 28.3 kcal mol^−1^), with the respective duplexes ranked as follows: CC**F**/G**F**G > **F**CC/G**F**G > C**F**C/G**F**G. As a consequence, the bistrand AP‐sites thermodynamically destabilize the parent tridecamer duplex with ΔΔG on the order of 7.1–8.2 kcal mol^−1^ and ranked as follows: CC**F**/G**F**G > **F**CC/G**F**G ∼ C**F**C/G**F**G. The net energetic impact is better visualized by comparing each bistrand AP‐damaged duplex with its corresponding single AP‐site counterpart as described below and illustrated graphically in Figure 3.

### 3.9 Differential energetic impact of introducing a second AP‐site within the CCC/GFG duplex

Comparisons between duplexes harboring **s**ingle and bistrand AP‐sites yield insight into the crosstalk between these lesions and overall impact of relative orientation as reflected in Table 2. The differential energetic impact of introducing a second abasic site within the CCC/G**F**G duplex yielding −1, 0, and +1 orientations is illustrated in Figure 3. Introduction of a second AP‐site in the *top* strand decreases the dissociation enthalpy by 11.4 kcal mol^−1^ for the 5′ staggered lesion (**F**CC/G**F**G) and 16.6 kcal mol^−1^ for the 3′ staggered lesion (CC**F**/G**F**G) relative to the single site CCC/G**F**G duplex with no further reductions observed for C**F**C/G**F**G (ΔΔ*H* = 0.2 kcal mol^−1^). Conversely, introduction of a second AP‐site in the top strand of CCC/G**F**G leads to a further reduction of the unfavorable entropic contribution to duplex stability by 10.8 kcal mol^−1^ for the 5′ staggered lesion (**F**CC/G**F**G) and 14.9 kcal mol^−1^ for the 3′ staggered lesion (CC**F**/G**F**G) with an increased entropic penalty observed for C**F**C/G**F**G (Δ*T*Δ*S* = 0.8 kcal mol^−1^). Reconciling the enthalpic and entropic impacts of bistrand abasic sites, there is a modest reduction in the association free energy of 0.7 kcal mol^−1^ (**F**CC/G**F**G) and 0.6 kcal mol^−1^ (C**F**C/G**F**G), with a substantial decrease in CC**F**/G**F**G (ΔΔ*G* = 1.8 kcal mol^−1^). In summary, we observe a threefold reduction in ΔG when a second AP‐site is introduced upstream from the central G**F**G lesion in CC**F**/G**F**G as compared to the **F**CC/G**F**G and C**F**C/G**F**G bistrand duplexes. Interestingly, the destabilizing impact of introducing two AP‐sites directly opposite each other in the C**F**C/G**F**G bistrand duplex is primarily entropic in origin. Relative to the corresponding single AP‐sites in top and bottom strands, the bistrand AP‐duplexes are destabilized with differential free energies (ΔΔ*G*s) ranked as follows: CC**F**/G**F**G > **F**CC/G**F**G ≥ C**F**C/G**F**G.

## 4 DISCUSSION

The overall interest in characterizing DNA duplex damage involving base substitution, insertion, or deletion mutations dates over three decades.^39^, ^40^, ^41^ During this timeframe, numerous investigations have explored the biochemical, biophysical, and structural impact of chemically modified bases, thereby providing insight regarding the molecular basis of lesion‐induced mutagenesis and cytotoxicity.^8^, ^10^, ^22^, ^24^, ^25^, ^26^, ^36^, ^38^, ^42^, ^43^, ^44^, ^45^, ^46^, ^47^, ^48^, ^49^, ^50^ A unique outcome of ionizing radiation and radiomimetic anticancer treatments is the induction of clustered damage in which two or more lesions are formed within a single DNA helical turn.^13^, ^14^, ^15^, ^16^ A common source of AP site formation in DNA is ionizing radiation arising from UV light exposure, which produces hydroxyl free radicals in the aqueous surroundings. These free radicals can interact with the DNA bases and cause deleterious effects such as spontaneous depurination.

Despite the relatively high efficiency of endonucleases in promoting strand scission at a single damaged site, the presence of proximate abasic sites in opposite strands may affect the ability of cellular repair machinery to process these damages.^20^ A single ionizing event exerts sufficient energy to create multiple hydroxyl free radicals, thus increasing the propensity to form clusters of AP sites within the same vicinity. Energy deposition by ionizing radiation can therefore lead to the formation of LMDS, including AP‐sites on opposite strands and often situated within a helical turn of DNA. Studies on clustered AP lesions have therefore been motivated by biochemical evidence that suggests the presence of two or more proximate lesions may impair repair enzyme activity.^12^

The experimental framework employs a standard reference duplex that has been explored in previous studies to characterize the energetic impact of single base adducts, bulges, and lesions.^8^, ^10^, ^22^, ^24^, ^25^, ^26^, ^36^, ^38^, ^42^, ^43^, ^44^, ^45^, ^46^, ^47^, ^48^, ^49^, ^50^, ^51^ These damaged bases are site‐specifically introduced within a tridecamer deoxyoligonucleotide duplex comprised of a defined core sequence designed to adopt a non‐self‐complementary bimolecular B‐DNA structure.^7^, ^8^, ^10^, ^38^, ^47^ The latter has been used as a model system for structural and biological studies on the impact of single and bistrand damage.^21^ Our longstanding goal is to establish correlations between thermodynamic, structural, and biological outcomes by employing similar sequence contexts and solution conditions, thereby minimizing the impact of model system variability.

### 4.1 Impact of abasic clustered lesions on DNA thermodynamic properties

Previous studies have employed a combination of model‐independent calorimetric and model‐dependent spectroscopic approaches to assess the impact of a single AP site on duplex thermal and thermodynamic properties.^8^ The resultant data reveal that abasic sites are thermally and thermodynamically destabilizing with an overall magnitude that is modulated by sequence context, flanking residues, and counterbase identity.^8^, ^9^, ^10^ Our observations are consistent with such studies in that a single AP‐site introduced at various positions within the central triplet of a parent CCC/GGG tridecamer duplex is thermally and thermodynamically destabilizing. These results serve as a baseline for our characterization of bistrand AP‐sites on DNA duplex energetics. Specifically, a centrally positioned AP‐site in G**F**G is retained as a common reference for the bistrand duplexes studied herein. Consequently, the overall impact of bistrand AP‐sites is assessed by comparing the energetics of duplexes harboring different lesion orientations with that of a single AP‐damaged CCC/G**F**G duplex (Figure 3).

We observe a consistent trend in which bistrand abasic duplexes are more destabilizing than their single AP‐site counterparts. Inspection of the energetic parameters reveals that bistrand abasic duplexes are destabilized relative to single AP‐site damage positioned in either the bottom (CCC/G**F**G) or top (i.e., CC**F**/GGG, **F**CC/GGG, and C**F**C/GGG) strands. Whereas thermodynamic destabilization of the 5′ **F**CC/G**F**G and 3′ CC**F**/G**F**G staggered duplexes is primarily enthalpic in origin, we find that a central C**F**C/G**F**G bistrand damaged duplex is destabilized entropically as illustrated in Figure 3. Our observation corroborates a prior study on C**F**C/G**F**G and a “parent” CC/GG dodecamer that exhibit comparable duplex dissociation enthalpies with the reduction in thermodynamic stability attributed to an entropic penalty.^7^ Our data therefore suggest that the presence of two opposing lesions might facilitate flanking residue stacking with solvent reorganization accounting for overall stability changes.

### 4.2 Melting cooperativity of single and bistrand AP‐damaged duplexes

An estimate of DNA duplex melting cooperativity relies on DSC analysis whereby the calorimetrically derived van't Hoff enthalpy is compared with that measured from the integrated area of the heat capacity profiles. Comparisons between calorimetrically (Δ*H*_cal_) and optically derived model dependent (Δ*H*_vH_) enthalpies may also provide information on the two‐state nature of DNA duplex dissociation. In the absence of calorimetric data, one useful approach involves comparison of optically derived van't Hoff enthalpies (Δ*H*_vH_) obtained via two distinct methods, namely shape analysis versus concentration dependence of the transition temperatures. A conventional criterion for assessing two‐state dissociation adopts a 15% cut‐off level for disparities between the two measurements.^33^ Inspection of the ratios between shape analysis and concentration dependent Δ*H*_vH_ summarized in Table 1 reveals that the single AP‐site **F**CC/GGG and CC**F**/GGG duplexes deviate from two‐state dissociation. Interestingly, the corresponding bistrand damaged duplexes (i.e., **F**CC/G**F**G and CC**F**/G**F**G) and C**F**C/G**F**G exhibit cooperativity ratios that are typical of two‐state melting. While these data await confirmation via DSC analysis, we might hypothesize that inclusion of a second AP‐site on the opposing strand (i.e., **F**CC/G**F**G and CC**F**/G**F**G) shifts the equilibrium from one that involves population of intermediate states to another that fits an “all‐or‐nothing dissociation process.”

Our finding that the three bistrand duplexes exhibit two‐state melting behavior provides assurance that the destabilization process is cooperative and specific. Moreover, these findings effectively eliminate the contribution of competing processes such as single strand equilibria, which normally precludes accurate evaluation of intrinsic lesion impacts.^25^, ^26^, ^38^ Extrapolations of melting data to 25°C employing Δ*C*_p_ are complicated by folding/self‐associating events coupled to the dissociation process. The absence of detectable single strand effects via the optical approaches employed herein affords an opportunity to estimate thermodynamic parameters at lower temperatures. While the **F**CC/GGG and CC**F**/GGG single site duplexes exhibit cooperativity ratios that are significantly <1, the three bistrand AP‐duplexes undergo a two‐state dissociation process as deduced from our analysis of temperature‐dependent hyperchromicity profiles. These findings validate our experimental approach and ensure the validity of applying a van't Hoff model for derivation of thermodynamic parameters.

### 4.3 Thermodynamic parameters at a standard reference state

A complete thermodynamic characterization of abasic site impacts on duplex energetics requires extrapolation of the data to a common reference temperature (e.g., 25°C). This necessitates specific knowledge of the heat capacity change (Δ*C*_p_) accompanying duplex to single strand transitions. The impact of single and bistrand abasic sites on Δ*C*_p_ has not been characterized sufficiently to date, particularly within the sequences employed herein. The optically derived energetic parameters are reported without explicit incorporation of heat capacity changes to facilitate comparison with thermodynamic data acquired on a number of other lesions and defects embedded within a similar sequence context.^8^, ^25^, ^26^, ^36^, ^38^, ^43^, ^44^, ^45^, ^46^, ^47^, ^51^ Nevertheless, we have evaluated the data at a common reference temperature of 25°C using an average Δ*C*_p_ ∼ 70 cal mol^−1^ bp^−1^ 52, 53 with the implicit understanding that future studies are required to corroborate this preliminary assessment. The energetic parameters reported in Supporting Information Table S2 reveal similar trends relative to the non heat capacity corrected data and corroborate the impact of a bistrand lesion on 3′‐orientation. Moreover, the unique behavior of centrally positioned bistrand AP‐sites is confirmed as reflected in the entropic term governing overall thermodynamic stability.

### 4.4 Nearest neighbor considerations

The parent tridecamer duplex exhibits thermodynamic properties expected for a fully associated canonical duplex. In fact, the measured dissociation enthalpy (i.e., Δ $H_{\text{vH}}^{\text{conc}}$ = 110.6 kcal mol^−1^) is consistent with its predicted value (i.e., Δ*H*_pred_ = 104.9 kcal mol^−1^) using nearest neighbor algorithms.^54^, ^55^, ^56^, ^57^ Although there are no algorithms available to predict the impact of lesions on duplex energetics, one may use values derived for canonical duplexes and evaluate whether the destabilization imparted by a lesion is local or propagates beyond the damaged site.^10^ As a case in point, we may hypothesize that the local impact of an AP‐site translates into perturbation of neighboring stacking interactions in addition to disruption of conventional Watson‐Crick base pairing. One might reasonably expect a destabilization enthalpy (i.e., ΔΔ*H*) on the order of 16–17 kcal mol^−1^ for a single abasic site positioned within the central CCC/GGG triplet of a tridecamer duplex.

Inspection of the enthalpic data for duplexes harboring single AP‐sites reveals that those situated 5′ (**F**CC/GGG) or 3′ (CC**F**/GGG) to the central base pair are generally consistent with expected local perturbations (i.e., ΔΔ*H* ≤ 17 kcal mol^−1^). Conversely, abasic sites centered within the parent tridecamer duplex (C**F**C/GGG and CCC/G**F**G) impart an enthalpic destabilization that is somewhat greater than those predicted for local perturbations (i.e., ΔΔ*H* = 18.3 and 19.9 kcal mol^−1^, respectively). The impact of bistrand AP‐sites is significantly more complex and cannot be evaluated simply in terms of nearest neighbor interactions. The destabilizing effect of bistrand AP‐sites requires further elaboration invoking potential energy coupling amongst these lesions. Structural evidence suggests that the replacement of one or two bases by AP‐sites does not simply leave a void but potentially creates opportunities for establishing new interactions between flanking bases.^21^ The latter may involve stacking of neighboring bases with their counterparts on the opposite strand, interactions that are not generally shared within an undamaged canonical duplex.

### 4.5 Structural implications

**Figure 4:**
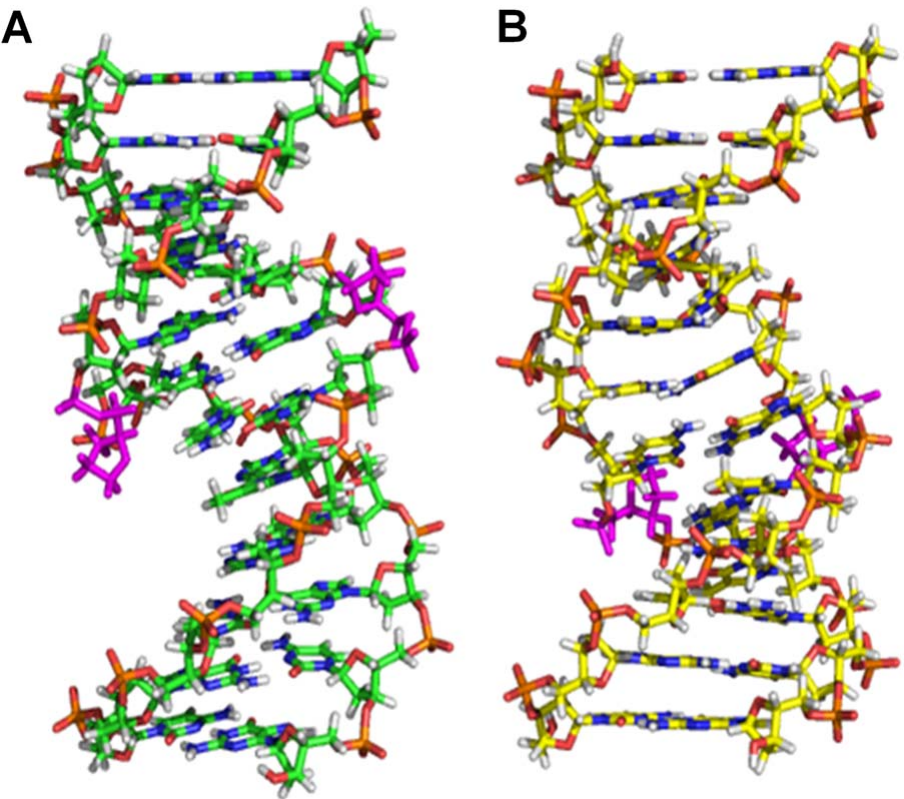
Structural Insights on Abasic DNA Constructs Deduced via NMR Characterization. A, The 5′‐staggered **F**CC/G**F**G duplex (PDB file 1YCT)^21^; B, The 3′‐staggered CC**F**/G**F**G duplex (PDB file 1YCW).^21^ These structures are prepared using PyMOL^63^ with AP‐sites (magenta) located in major and minor grooves of the **F**CC/G**F**G and CC**F**/G**F**G duplexes, respectively

The impact of bistrand AP‐sites centered within a similar sequence context as that employed herein has been evaluated by NMR and restrained molecular dynamics.^21^ The structural impact of staggered AP‐lesions positioned 5′ or 3′ to each other is illustrated in Figure 4. These findings suggest that the relative orientation of two abasic sites located within a single helical turn exerts a profound impact on DNA conformation and enzyme repair efficiency. Although structural preferences are presumably responsible for the differential susceptibility of staggered lesions to repair enzyme processing, the thermodynamic origins of such impacts have not been fully characterized to date. Our study is designed to address this deficiency by evaluating global structural properties within the context of DNA thermodynamic stability in the absence and presence of one or two AP lesions positioned in opposite strands.

NMR studies on the parent tridecamer DNA duplex dGCGTACCCATGCG·dCGCATGGGTACGC harboring bistrand AP‐sites within the central CCC/GGG triplet reveal that both lesions in **F**CC/G**F**G *bulge out* of the duplex, thereby allowing preferential binding between the adjacent C_20_ and G_6_.^21^ While the AP‐sites in **F**CC/G**F**G reside entirely outside the duplex and form abasic bulges, the AP sites in CC**F**/G**F**G (i.e., 3′‐staggered) extrude unequally from the helix. The AP‐site neighbored by a guanine forms an abasic bulge, while that neighbored by a cytosine aligns with the backbone. The resultant structural effect is that an adenine phosphate backbone is extruded towards the solvent.^21^ NMR structures of these staggered conformations reveal that the 3′ CC**F**/G**F**G duplex is more structurally perturbed than its 5′ **F**CC/G**F**G counterpart. Moreover, abasic lesions in the CC**F**/G**F**G duplex reside in the minor groove, while AP sites in **F**CC/G**F**G are located within the major groove.^21^ These solution structural studies are consistent with our thermodynamic data in that the 3′‐staggered bistrand abasic duplex is more destabilized relative to its 5′‐counterpart. Collectively, structural and thermodynamic data appear to isolate CC**F**/G**F**G as the most impairing configuration among the bistrand duplexes studied herein.

A dearth of NMR studies on the C**F**C/G**F**G configuration effectively precludes correlation of structural‐energetic impacts for such bistrand duplexes. Nevertheless, a recent report has addressed the structural and dynamic impacts of AP‐clustered duplexes at various inter‐lesion distances and orientations including abasic sites positioned directly opposite each other.^34^ Remarkably, these AP‐sites are fully extruded as a direct consequence of flanking residue stacking that “shrinks” the helix and narrows the minor groove. These observations are consistent with the entropic penalty measured for bistrand relative to single AP‐site duplexes, as flanking residue stacking may effectively prevent further enthalpic losses, albeit at the expense of decreasing lesion‐induced conformational flexibility. The implications of these findings to lesion recognition and repair are discussed in the following section.

### 4.6 Implications for AP‐recognition and repair

Previous studies have reported that clusters of lesions can affect BER efficiency in a manner that depends on the distance and orientation of AP‐sites in opposing strands.^12^, ^14^, ^17^, ^20^, ^21^, ^34^ In the case of lesions positioned a single base apart in opposite strands, there is a preference for processing those situated 3′ *in lieu of* 5′ or directly opposed to each other.^21^, ^34^ As a case in point, hAPE 1 and *E. coli* Nfo incise +1 clusters reasonably well, while −1 bistrand lesions are processed at significantly reduced rates relative to a single AP‐site incision. Double‐strand breaks are generated in duplexes with abasic sites positioned 3′ to each other, similar to the sequence of our 5′‐staggered **F**CC/G**F**G bistrand lesions. In duplexes containing AP sites positioned a single base pair apart and 5′ to each other, both APE and exonuclease III slowly cleave the abasic site on one strand yet are unable to incise AP on the opposite strand.^20^ Studies conducted on identical sequences as those employed herein reveal that both abasic sites within **F**CC/G**F**G are readily processed by APE. This observation contrasts with the CC**F** strand of CC**F**/G**F**G in which the AP‐site is refractory to APE incision.^21^

Despite the general consensus that repair enzymes sense lesions within destabilized regions,^22^, ^44^ there is a delicate balance between energetics and the ability to form an adequate enzyme‐substrate arrangement given the requirement for a minimum local duplex stability. Although greater flexibility may facilitate access of the enzyme to a lesion site, the local geometry must be maintained. Four of the duplexes studied herein harbor single AP‐sites that are positioned either opposite G (**F**CC/GGG, C**F**C/GGG, and CC**F**/GGG) or opposite C (CCC/G**F**G). While there is evidence that specific repair machineries are modulated exquisitely by the identity of a lesion counterbase, studies suggest that AP‐endonuclease recognizes and processes abasic sites in a counterbase‐independent manner.^23^ It is therefore likely that the differential APE processing efficiency arises from lesion spatial orientation and the underlying energetics, as opposed to counterbase preferences. The two 5′ and 3′‐staggered bistrand AP‐duplexes studied herein are processed by APE with distinct efficiency. Specifically, AP lesions in the 3′‐orientation (CC**F**/G**F**G) are more refractory to enzyme processing than those in the 5′‐orientation (**F**CC/G**F**G).^21^ The authors present a compelling argument for structural discrimination between these two duplexes despite the absence of thermal stability differences. In contrast with the findings of NMR assessments, our combined temperature‐dependent UV and CD spectroscopic data clearly demonstrate that these bistrand duplexes exhibit significant thermodynamic differences. In fact, the 3′‐bistrand CC**F**/G**F**G duplex exhibits the lowest thermodynamic stability amongst all duplexes studied.

These observations might seem counterintuitive considering the general consensus that cellular repair machinery recognizes target lesions by sensing structural and energetic perturbations. Consequently, highly destabilized duplexes are presumably processed with greater efficiency via energetic recognition mechanisms.^22^, ^44^ It is possible that the greater destabilization imparted by a 3′‐AP bistrand lesion renders the local helical conformation poised to undergo transient dissociation, thereby populating a species of single stranded states. Supporting this hypothesis, we find that lesions are generally repaired within fully associated duplexes while single stranded sequences are much less efficiently recognized and/or repaired by enzyme systems. In the case of APE, reports suggest that this enzyme is capable of processing AP‐sites within single strands,^58^ yet the presence of monomolecular self‐structures populated transiently during enzyme assays has not been effectively excluded. Specifically, staggered AP‐sites positioned opposite G, T, and to some extent C (but not A) may undergo transient local melting at ambient temperatures,^5^ which might serve as a sensor for the repair machinery. Conversely, excessive destabilization might actually impair enzyme recognition in the case of specific bistrand lesion orientations such as CC**F/**G**F**G.

Bistrand abasic lesions positioned directly opposite one another (C**F**C/G**F**G) are implicated as representing a unique challenge to the repair machinery^23^ and are highly mutagenic.^6^ In this particular case, one might logically conclude that both AP‐sites are extruded from the helix, thereby allowing stacking of the flanking residues. Considering the unique structural properties of C**F**C/G**F**G, we might gain significant insight by comparing the bistrand duplex energetics with that of a canonical dodecamer harboring an identical sequence that has been characterized in our laboratory.^7^ Indeed, “insertion” of two opposing AP‐sites into an undamaged dodecamer duplex reduces the free energy by 2.5 kcal mol^−1^, which is the result of an entropic penalty with virtually unchanged enthalpy.^7^ In this study, we have compared thermodynamic profiles of bistrand duplexes with their corresponding single AP‐site counterparts. Remarkably, the 5′ **F**CC/G**F**G and 3′ CC**F/**G**F**G bistrand duplexes are destabilized enthalpically relative to CCC/G**F**G, whereas the C**F**C/G**F**G bistrand duplex is destabilized entropically relative to its respective single AP‐site “precursors” as documented in Table 2 and Figure 3. It is relevant to note that studies exploring the impact of lesions on DNA duplex energetics and stability are influenced by an array of solution conditions and variables including ionic strength, pH, and sequence context. As a specific example, one observes a significant sequence context‐dependence when comparing the processing efficiency of excising AP‐sites in CpG islands versus TATA‐box‐like sequences.^59^ These findings represent a cautionary warning that studies employing specific sequences might not necessarily reflect an accurate overall outcome and generalizations must include a requisite caveat.

### 4.7 Biological implications

A wealth of evidence suggests that the cellular repair machinery “senses” structural and thermodynamic perturbations that are associated with the presence of a damaged base.^22^, ^44^ Assuming that this is the probing mechanism of endonucleases to recognize AP‐sites, characterization of AP‐lesion impacts on DNA structure and stability provides insights on the origins of AP‐recognition and excision. Considering the additional challenge faced by repair systems in the presence of clustered AP‐lesions, a direct correlation of lesion‐induced destabilization versus repair enzyme efficiency might represent an oversimplification. One observes a relatively high occurrence of AP‐lesion damage as ∼5,000–10,000 sites appear per cell on a daily basis.^60^ Exposure to ionizing radiation such as γ‐ and X‐rays incurred via radiotherapy may lead to the accumulation of two or more AP‐sites within a few DNA helical turns.

These findings portend the urgency of undertaking systematic investigations to characterize isolated and clustered lesion impacts on the biophysical, functional, and structural properties of host DNA duplexes. The acquisition of multidisciplinary databases advances our understanding and improves structural, energetic, and functional correlations needed to intervene in the repair processes that occur within normal and cancer cells. While APE processing efficiency is fundamental in maintaining normal cell genome stability, ionizing radiation targets cancer cells by creating LMDS that eventually overcome enzyme repair capacity. The design of small molecules that synergistically interact with and inhibit lesion cluster repair may advance anticancer therapeutic interventions. Understanding the energetic basis of APE recognition paves the way for characterizing repair selectivity towards clustered AP‐lesions and identifying rational approaches to modulate these intricate cellular processes.

## 5 CONCLUDING REMARKS

Thermodynamic analysis of temperature‐dependent UV melting transitions in conjunction with structural assessment of CD profiles reveals that the relative orientation of abasic sites in bistrand‐damaged duplexes imparts a significant energetic impact. Specifically, 3′ oriented lesions (CC**F**/G**F**G) within the central triplet of a tridecamer duplex exert a greater destabilizing effect when compared to their 5′ counterpart (**F**CC/G**F**G). Remarkably, bistrand lesions positioned opposite each other (C**F**C/G**F**G) exhibit minimal enthalpic changes yet are destabilized entropically relative to single AP‐site duplexes. Collectively, our energetic data are generally consistent with the structural properties of bistrand AP‐site duplexes in which a 3′ orientation exerts a greater perturbation when compared to the corresponding 5′ lesion.^21^ These findings provide additional insight regarding cellular repair enzyme efficiency as bistrand AP lesions in a 5′ staggered orientation are processed more effectively.^21^, ^61^, ^62^

## 6 DEDICATION

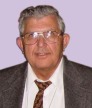

We dedicate this manuscript to our beloved friend and cherished colleague Jack Aviv, whose personal and scientific legacy will always remain an enduring and inspiring beacon to both current and future generations of scientists. As the Founder and President of Aviv Biomedical, Jack established an unparalleled reputation in the field of spectroscopy by designing and fabricating state‐of‐the‐art analytical instrumentation that has allowed researchers to explore new frontiers at the interface of biology, chemistry, and medicine. Widely acknowledged amongst his peers as an expert and leader in the field, Jack's contributions to spectroscopic characterization of biological macromolecules are innumerable and legendary. We fondly remember Jack as an innovator, mentor, and philanthropist who forged lifelong friendships within the scientific community. Jack's philanthropic nature is evident in his longstanding support of The Calorimetry Conference, personally funding the William F. Giauque and Stig Sunner Memorial Awards that highlight the achievements of aspiring young investigators. In recognition of his distinguished lifetime service, The Calorimetry Conference convened the Jack Aviv Biothermodynamics Symposium at its Seventy Second Annual Meeting during the week of 30 July–03 August 2017 in Colorado Springs, Colorado. Florence Aviv, Debi Aviv Bialis, Matthew Bialis, and Daniel Aviv served as Honorary Symposium Co‐Chairs, longtime friends and colleagues contributed Invited Lectures, and The Calorimetry Conference conferred the inaugural Jack Aviv Memorial Award to Kenneth J. Breslauer who presented the Keynote Address. We are particularly indebted to Jack for his active role in nurturing and recruiting young scientists at the outset of their professional careers. This manuscript reflects Jack's true passion in training future generations of biophysicists as the structural and thermodynamic data reported herein have been acquired on Aviv Biomedical CD and UV/Vis spectrophotometers by three talented undergraduate students who are co‐authors on this study. Spanning over four decades of an abiding friendship that will be treasured forever, we gratefully acknowledge the indelible contributions of Jack Aviv and dedicate this manuscript to our dearest friend with the deepest admiration, love, and respect.

## Supporting information

Additional Supporting Information may be found online in the supporting information tab for this article.

Supporting Information
