# Supplementary material for: Impact of bistrand abasic sites and proximate orientation on DNA global structure and duplex energetics
Source: Biopolymers. 2018 Jan 11;109(8):e23098. doi: 10.1002/bip.23098 (PMC6175389; doi:10.1002/bip.23098)
Supplement: Supplementary file 1 — Supporting Information [file BIP-109-na-s001.pdf]

# SUPPORTING INFORMATION

## Impact of Bistrand Abasic Sites and Proximate Orientation on DNA Global Structure and Duplex Energetics

Conceição A. Minetti, Jeffrey Y. Sun, Daniel P. Jacobs, Inkoo Kang,
David P. Remeta and Kenneth J. Breslauer ✉

*Rutgers - The State University of New Jersey*
*Department of Chemistry and Chemical Biology*
*Piscataway, New Jersey 08854*

TABLE S1. Summary of van't Hoff Thermodynamic Parameters derived via Shape Analysis of Temperature-dependent Profiles acquired for the Tridecameric Duplexes.............. 2

TABLE S2. Heat Capacity corrected Thermodynamic Dissociation Parameters. ............................ 3

FIGURE S1. Thermal Stability of Canonical, Single, and Bistrand AP-Site Duplexes..................... 4

FIGURE S2. Dissociation Enthalpies of Canonical, Single, and Bistrand AP-Site Duplexes. .......... 5

FIGURE S3. Dissociation Entropies of Canonical, Single, and Bistrand AP-Site Duplexes. ........... 6

FIGURE S4. Dissociation Free Energies of Canonical, Single, and Bistrand AP-Site Duplexes. ..... 7

***TABLE S1. Summary of van't Hoff Thermodynamic Parameters derived via Shape Analysis of Temperature-dependent Profiles acquired for the Tridecameric Duplexes.***

| **Acronym** | **$\Delta H^o_{shape}$ (kcal·mol$^{-1}$)** | **$T\Delta S^o_{shape}$ (kcal·mol$^{-1}$)** | **$\Delta G^o_{shape}$ (kcal·mol$^{-1}$)** |
|---|---|---|---|
| **CCC/GGG** | **113.7** | **91.1** | **22.5** |
| **FCC/GGG** | **69.9** | **56.7** | **13.2** |
| **CFC/GGG** | **86.7** | **72.2** | **14.5** |
| **CCF/GGG** | **70.8** | **57.6** | **13.2** |
| **CCC/GFG** | **79.0** | **65.1** | **13.9** |
| **FCC/GFG** | **85.8** | **71.2** | **14.5** |
| **CFC/GFG** | **81.9** | **68.2** | **13.6** |
| **CCF/GFG** | **77.0** | **64.1** | **12.9** |

Thermodynamic data correspond to a DNA duplex of 45.4 μM total strand concentration ($C_T$).
Standard errors for $\Delta G^o$, $\Delta H^o$, and $T\Delta S^o$ are within 2.0, 4.5, and 5.0 kcal·mol$^{-1}$, respectively.

***TABLE S2. Heat-Capacity Corrected Thermodynamic Dissociation Parameters.***

| Acronym | $\Delta H°_{25\,°C}$ (kcal·mol$^{-1}$) | $T\Delta S°_{25\,°C}$ (kcal·mol$^{-1}$) | $\Delta G°_{25\,°C}$ (kcal·mol$^{-1}$) |
|---|---|---|---|
| **CCC/GGG** | **79.32** | **63.3** | **16.0** |
| **FCC/GGG** | **80.56** | **69.2** | **11.4** |
| **CFC/GGG** | **74.16** | **63.4** | **10.8** |
| **CCF/GGG** | **76.07** | **65.1** | **10.9** |
| **CCC/GFG** | **72.51** | **61.8** | **10.7** |
| **FCC/GFG** | **60.65** | **50.7** | **10.0** |
| **CFC/GFG** | **74.20** | **63.9** | **10.3** |
| **CCF/GFG** | **58.55** | **49.6** | **8.9** |

Values extrapolated to 25 ºC using a $\Delta C_p$ of 780 cal·mol$^{-1}$·deg $^{-1}$ (i.e., 60 cal·mol$^{-1}$·deg $^{-1}$·bp$^{-1}$) applying the following relations: $\Delta H\ (T) = \Delta H\ (T_m) – \Delta C_p\ (T_m – T)$; $\Delta S\ (T) = \Delta H/T_m + \Delta C_p\ ln(T/T_m) + R\ ln\ (C_T/4)$; $\Delta G\ (T) = \Delta H(T_m)\ (1 - T/T_m) + \Delta C_p\ [T - T_m - T\ ln\ (T/T_m)] - RT\ ln\ (C_T/4)$, *whereby T = 25 ºC.*

***FIGURE S1. Thermal Stability of Canonical, Single, and Bistrand AP-Site Duplexes.***

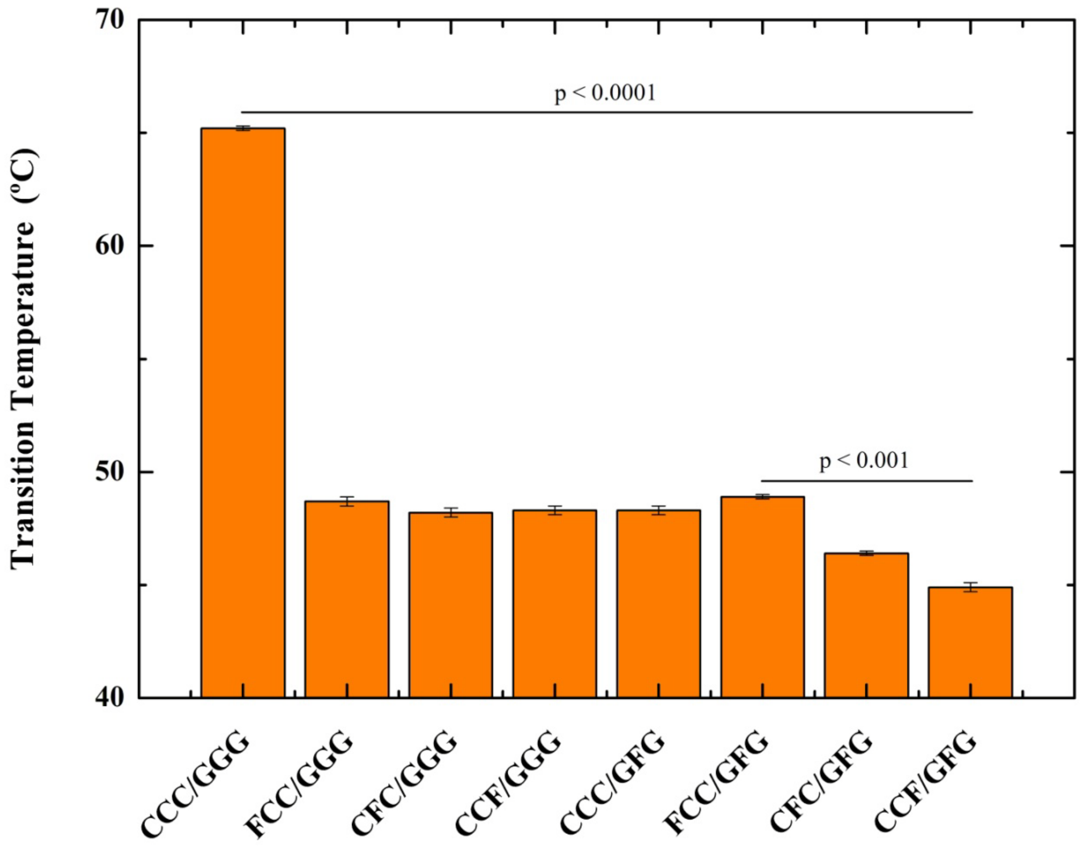


The single and bistrand abasic site duplexes exhibit statistically significant differences in thermal stability ($p < 0.0001$) relative to the undamaged parent tridecamer. The differential thermal stability of CFC/GFG and CCF/GFG are statistically significant ($p < 0.001$) when compared to the corresponding single AP-site reference duplexes CFC/GGG and CCF/GGG The bistrand CFC/GFG and CCF/GFG duplexes are thermally destabilized with CCF/GFG > CFC/GFG ($p < 0.001$). Thermal destabilization of FCC/GFG does *not* differ significantly ($p > 0.5$) relative to the single AP-site CCC/GFG and FCC/GGG duplexes.

***FIGURE S2. Dissociation Enthalpies of Canonical, Single, and Bistrand AP-Site Duplexes.***

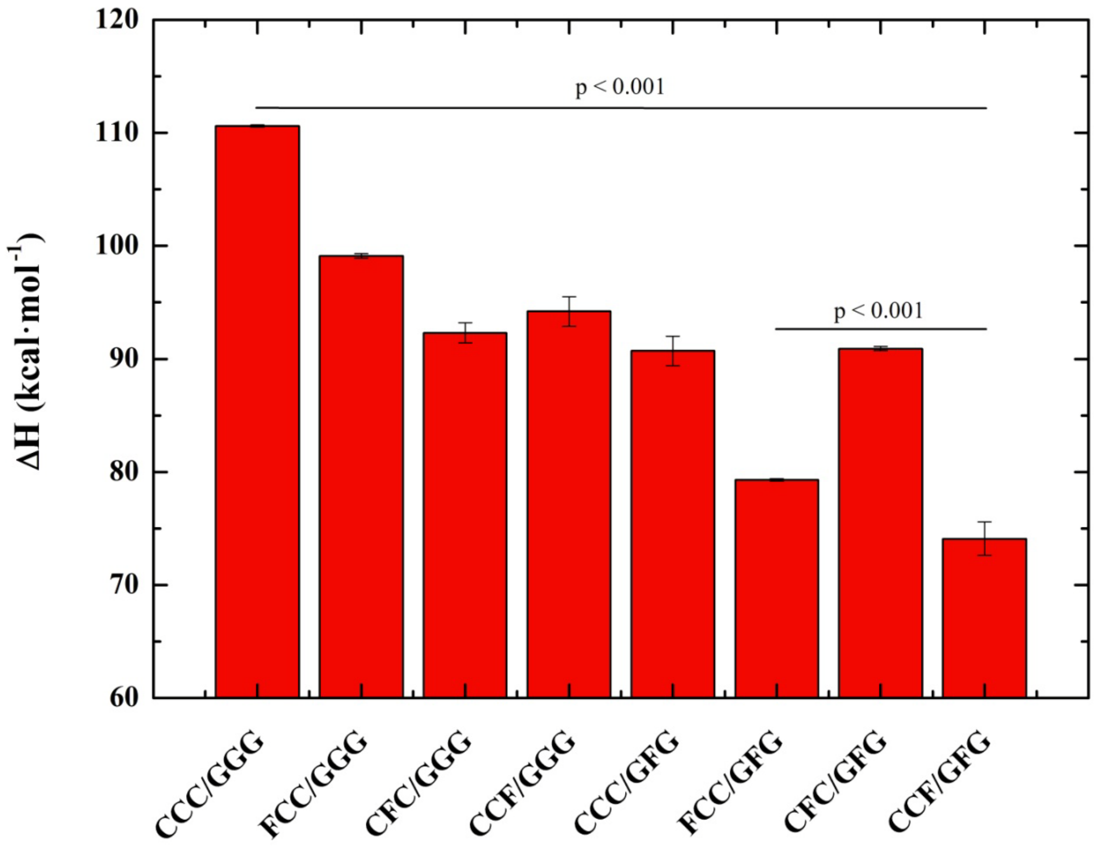


The single and bistrand abasic site duplexes exhibit statistically significant differences in enthalpic stability ($p < 0.001$) relative to the undamaged parent tridecamer. The differential enthalpic stability of FCC/GFG and CCF/GFG are statistically significant ($p < 0.001$) when compared to the corresponding single AP-site reference duplexes FCC/GGG and CCF/GGG. Enthalpic destabilization of the bistrand FCC/GFG and CCF/GFG duplexes are comparable ($p > 0.5$). The dissociation enthalpy of CFC/GFG does *not* differ significantly ($p > 0.5$) relative to the single AP-site CCC/GFG and CFC/GGG duplexes.

***FIGURE S3. Dissociation Entropies of Canonical, Single, and Bistrand AP-Site Duplexes.***

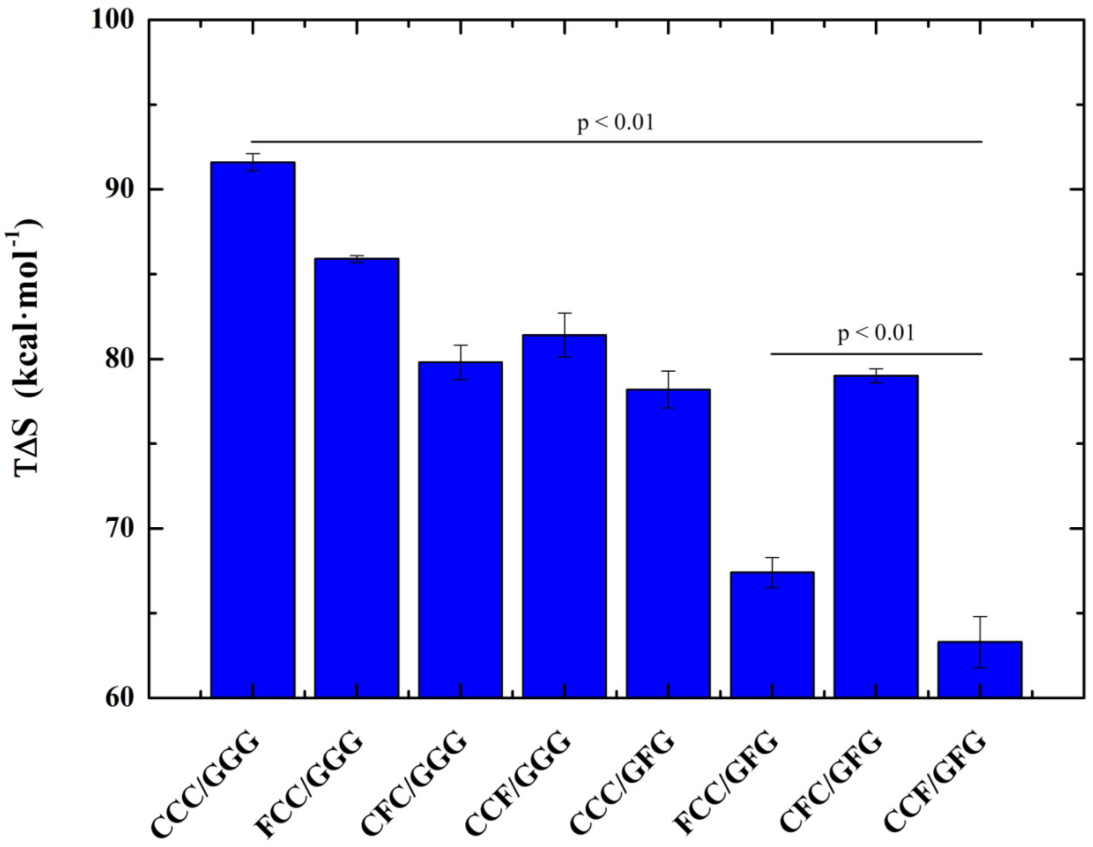


The single and bistrand abasic site duplexes exhibit statistically significant differences in entropic stability ($p < 0.01$) relative to the undamaged parent tridecamer. The differential entropic stability of FCC/GFG and CCF/GFG are statistically significant ($p < 0.001$) when compared to the corresponding single AP-site reference duplexes FCC/GGG and CCF/GGG. Entropic stabilization of the bistrand FCC/GFG and CCF/GFG duplexes are comparable ($p > 0.5$). The dissociation entropy of CFC/GFG is *not* significantly different ($p > 0.5$) relative to the single AP-site CCC/GFG and CFC/GGG duplexes.

***FIGURE S4. Dissociation Free Energies of Canonical, Single, and Bistrand AP-Site Duplexes.***

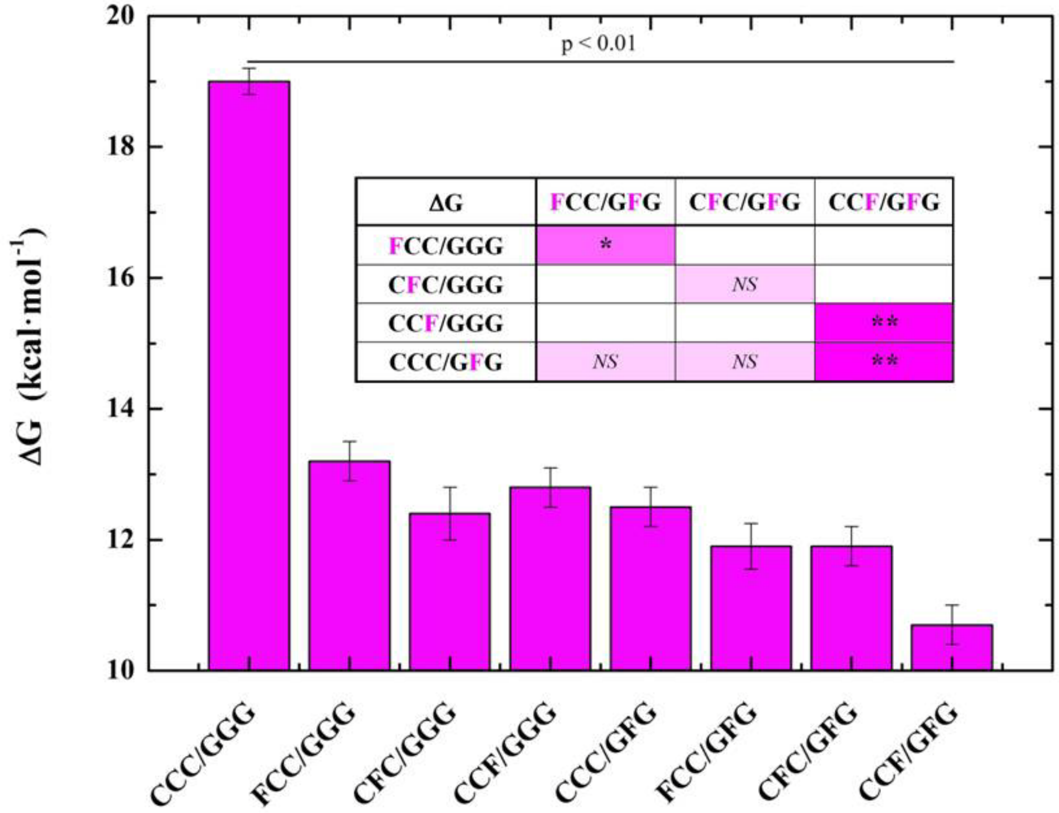


The single and bistrand abasic duplexes exhibit statistically significant differences in thermodynamic stability ($p < 0.01$) relative to the undamaged parent tridecamer. INSET: Statistical significance of dissociation free energy (ΔG) differences employing ANOVA and Tukey tests. Meaningful comparisons are designated as ∗∗ and ∗ signifying that the corresponding means differ at a level of $p < 0.05$ and $p < 0.1$, respectively. An *NS* designation indicates that the values differ somewhat albeit not significantly.
